# Predictive ability of visit-to-visit blood pressure indices for adverse events in patients with non-valvular atrial fibrillation: Subanalysis of the J-RHYTHM Registry

**DOI:** 10.1016/j.ijcha.2023.101216

**Published:** 2023-05-06

**Authors:** Eitaro Kodani, Hiroshi Inoue, Hirotsugu Atarashi, Ken Okumura, Shinya Suzuki, Takeshi Yamashita, Hideki Origasa, on behalf of the J-RHYTHM Registry Investigators

**Affiliations:** a Department of Cardiovascular Medicine, Nippon Medical School Tama Nagayama Hospital, Tokyo, Japan; b Saiseikai Toyama Hospital, Toyama, Japan; c AOI Hachioji Hospital, Tokyo, Japan; d Saiseikai Kumamoto Hospital, Kumamoto, Japan; e The Cardiovascular Institute, Tokyo, Japan; f The Institute of Statistical Mathematics, Tokyo, Japan

**Keywords:** Atrial fibrillation, Visit-to-visit blood pressure, Variability, Consistency, Adverse events

## Abstract

**Background:**

We previously reported that standard deviation (SD) of systolic blood pressure (SBP), an index of BP variability, and SBP-time in target range (TTR), an index of BP consistency, were significantly associated with adverse events in patients with non-valvular atrial fibrillation (NVAF). Thus, this study aimed to compare predictive ability for adverse events among visit-to-visit BP variability/consistency indices using data from the J-RHYTHM Registry.

**Methods:**

Of 7406 outpatients with NVAF, 7226 (age, 69.7 ± 9.9 years; men, 70.7%), in whom BP was measured 4 times or more (14.6 ± 5.0 times) during the 2-year follow-up period or until occurrence of an event, were included. As BP consistency for target SBP between 110 and 130 mmHg, SBP-TTR by the Rosendaal method and SBP-frequency in range (FIR) were calculated. Predictive ability was expressed by the area under receiver-operating-characteristic curve (AUC). AUCs of SBP-TTR and SBP-FIR for adverse events were compared with those of SBP-SD by the DeLong’s test.

**Results:**

SBP-SD, SBP-TTR, and SBP-FIR were 11.0 ± 4.2 mmHg, 49.5 ± 28.3%, and 52.3 ± 23.0%, respectively. AUCs of these indices for thromboembolism, major hemorrhage, and all-cause death were 0.62, 0.64, and 0.63 for SBP-SD; 0.56, 0.55, and 0.56 for SBP-TTR; and 0.55, 0.56, and 0.58 for SBP-FIR; respectively. AUCs of SBP-SD were significantly larger than those of SBP-TTR for major hemorrhage (P = 0.010) and all-cause death (P = 0.014), and SBP-FIR for major hemorrhage (P = 0.016).

**Conclusion:**

Among visit-to-visit BP variability/consistency indices, predictive ability of SBP-SD for major hemorrhage and all-cause death was superior to that of SBP-TTR and SBP-FIR in patients with NVAF.

## Highlights

• Systolic blood pressure (SBP)-standard deviation (SD) was superior to SBP-time in target range for predicting major hemorrhage and all-cause death.
• SBP-SD was superior to SBP-frequency in range for predicting major hemorrhage.
• BP variability is a more powerful indicator of adverse events than BP consistency in patients with non-valvular AF.

## Graphical abstract

AF, atrial fibrillation; BP, blood pressure; SBP, systolic blood pressure; SD, standard deviation; TTR, time in target range; FIR, frequency in range; ROC, receiver operating characteristic.

**Figure:**
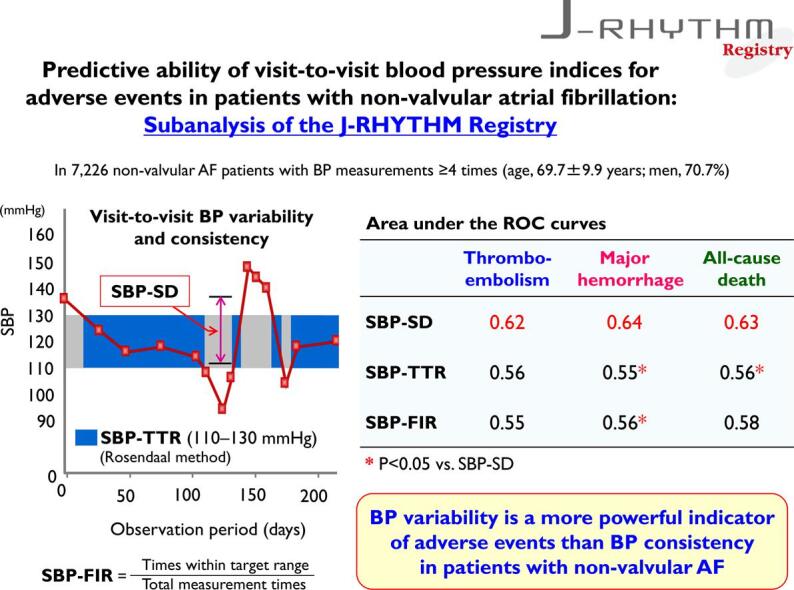

## 1 Introduction

Hypertension is a well-known risk factor for cardiovascular diseases [1], [2], [3], [4]. In patients with atrial fibrillation (AF), higher blood pressure (BP) levels were reportedly associated with an increased risk of thromboembolism and major hemorrhage [5], [6], [7], [8], [9]. Among substudies on hypertension or BP of the phase III clinical trials using non-vitamin K antagonist oral anticoagulants (NOACs), baseline BP values [6], [7], [8] and mean BP values [5], [9] were used to investigate the association with adverse events. BP values at any time during the follow-up period were adopted in only one study [9]. Accordingly, it remains uncertain which BP value is suitable for risk prediction in patients with AF.

We previously reported that baseline BP values did not emerge as an indicator of thromboembolism, but higher systolic BP (SBP ≥ 136 mmHg) at the time closest to an event or at the last visit of follow-up (SBP-end) were significantly associated with an increased risk of thromboembolism and major hemorrhage in patients with non-valvular AF (NVAF) [10]. Among visit-to-visit BP indices, SBP-standard deviation (SD), an index of long-term BP variability [11], was independently associated with the incidence of thromboembolism, major hemorrhage, and all-cause death [12]. We further demonstrated that SBP-time in target range (TTR), an index of BP consistency during the follow-up period, was also associated with cardiovascular death [13] from the J-RHYTHM Registry. Thus, this study aimed to compare predictive ability for adverse events among visit-to-visit BP variability/consistency indices using data from the J-RHYTHM Registry. In addition, we identified factors associated with visit-to-visit BP variability/consistency among baseline patient characteristics and medications.

## 2 Methods

### 2.1 *Study design of the J-RHYTHM Registr*y

The J-RHYTHM Registry was a nationwide prospective observational study to investigate the status of anticoagulation therapy and the optimal anticoagulation therapy in Japanese patients with AF [14]. The study design and patient characteristics at the time of enrollment were reported elsewhere [14], [15]. Briefly, a consecutive series of outpatients with AF was recruited from 158 institutions in 2009, regardless of antihypertensive drug use. All drugs and their dosages were determined at the discretion of the treating physicians. Patients with valvular AF defined as those with mitral stenosis or mechanical prosthetic valves were excluded from this subanalysis. Brachial BP was measured in a sitting position at the time of enrollment (baseline) and at each visit during the follow-up period by the auscultatory method or an automated sphygmomanometer, as appropriate at each institution, regardless of its vender [10], [12], [13]. The study protocol conformed to the Declaration of Helsinki and was approved by the ethics committee of each participating institution. Written informed consent was obtained from all participants at the time of enrollment.

For the present *post hoc* analysis, patients with NVAF, in whom BP was measured 4 times or more during the 2-year follow-up period or until occurrence of an event, were included based on the previous studies [12], [13], [16]. The primary endpoints were defined as follows: thromboembolism including symptomatic ischemic stroke, transient ischemic attack (TIA), and systemic embolism; major hemorrhage including intracranial hemorrhage, gastrointestinal hemorrhage, and other hemorrhages requiring hospitalization; and all-cause and cardiovascular death. The diagnostic criteria for each event have been described elsewhere [14], [15].

### 2.2 Evaluation of visit-to-visit BP variability/consistency indices and predictive ability for adverse events

Among visit-to-visit BP variability/consistency indices, BP variability was evaluated by SBP-SD during the follow-up period as in previous studies [11], [12], [16]. Since the usefulness of SBP-coefficient of variation (CV = SD/mean) for predicting adverse events were comparable with that of SBP-SD in our previous report using the same study cohort [12], only SBP-SD was adopted as the BP variability index in this subanalysis.

The overall BP consistency during the follow-up period was evaluated by 2 methods. First, SBP-TTR was calculated using a linear interpolation method by Rosendaal [17]. This method is analogous to the time in therapeutic range of prothrombin time international normalized ratio (PT-INR) and widely used to evaluate the long-term quality of anticoagulation therapy in patients receiving warfarin [18], [19]. Second, SBP-frequency in range (FIR) was obtained from the formula (FIR = [times within target range]/[total measurement times]) [20]. This method is easily employed for the evaluation of BP consistency in a clinical setting because the FIR can be calculated without a complex formula. In this study, the target SBP range was set between 110 and 130 mmHg since this range was better for evaluation of the association with adverse events than that of 120–140 mmHg in our previous report using the same study cohort [13]. Predictive ability of these SBP variability/consistency indices for adverse events was expressed by the area under the receiver-operating-characteristic (ROC) curve (AUC).

### 2.3 Statistical analyses

Data are presented as mean ± SD or number (percentage). AUCs of SBP-TTR and SBP-FIR for adverse events were compared with those of SBP-SD by the DeLong's test [21]. To identify factors associated with visit-to-visit BP variability and consistency among baseline patient characteristics and medications, simple regression analysis was performed first. Subsequently, multiple regression analysis with stepwise forward procedure was performed using significant variables in simple regression analysis. Statistical analyses were performed with SPSS software version 23.0 (IBM Corporation, Armonk, NY, USA) and EZR version 1.54 [22] on R version 4.0.3 (The R Foundation for Statistical Computing, Vienna, Austria). Two-tailed P-values < 0.05 were considered statistically significant.

## 3 Results

Of the 7937 entire patients with AF enrolled in the J-RHYTHM Registry [15], 421 (5.3%) with valvular AF were excluded and 110 (1.5%) were lost to follow-up. Of the remaining 7406 patients with NVAF [10], [23], 180 (2.4%) patients with BP measurements<4 times during the follow-up period were excluded. Consequently, 7226 patients (age, 70 ± 10 years; men, 71%) were included in this subanalysis [12], [13].

### 3.1 Baseline patient characteristics and medications

**Fig. 1:**
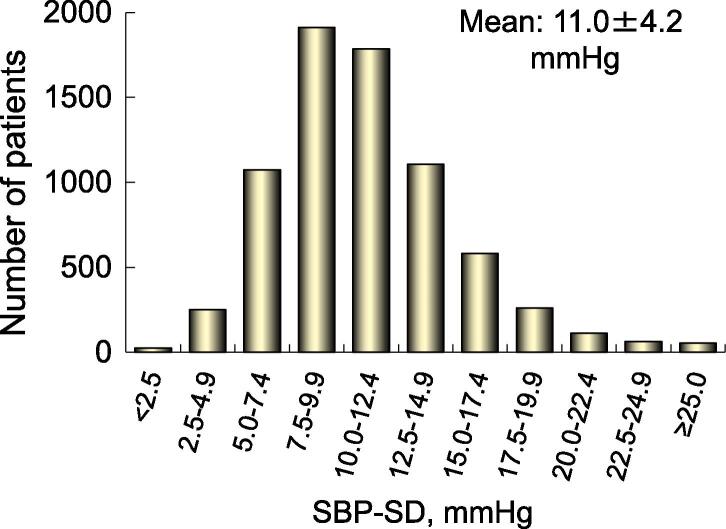
Distributions of SBP-SD. SBP, systolic blood pressure; SD, standard deviation.

**Fig. 2:**
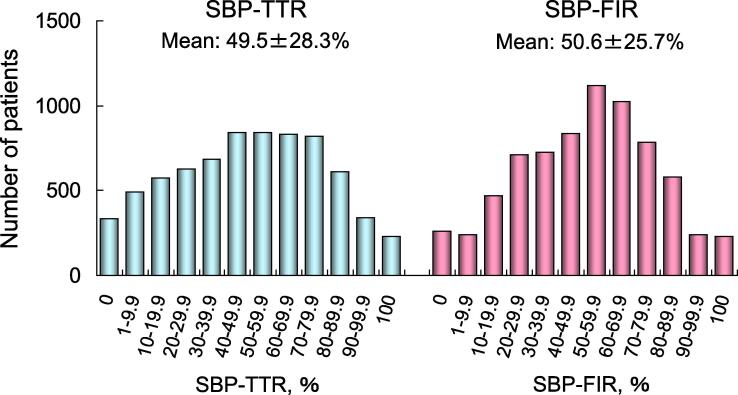
Distributions of SBP-TTR and SBP-FIR. SBP, systolic blood pressure; TTR, time in target range; FIR, frequency in range. Target SBP was 110–130 mmHg.

Clinical characteristics and medications of 7226 patients are listed in Table 1. Approximately 60% of the patients had hypertension, and BP was measured 14.6 ± 5.0 times during the follow-up period. SBP-SD, SBP-TTR, and SBP-FIR were 11.0 ± 4.2 mmHg, 49.5 ± 28.3%, and 51.3 ± 28.5%, respectively (Table 1). Distributions of these indices are shown in Fig. 1, Fig. 2.

**Table 1:** Patient characteristics and medications.

Number of patients	7226
Age, years	69.7 ± 9.9
Sex, male	5108 (70.7%)
Body mass index, kg/m^2^ (n = 6242)	23.6 ± 4.0
Type of atrial fibrillation	
Paroxysmal	2762 (38.2%)
Persistent	1056 (14.6%)
Permanent	3408 (47.2%)
Comorbidities	
Coronary artery disease	755 (10.4%)
Cardiomyopathy	620 (8.6%)
Hypertrophic cardiomyopathy	258 (3.6%)
Dilated cardiomyopathy	362 (5.0%)
Congenital heart disease	96 (1.3%)
COPD	124 (1.7%)
Hyperthyroidism	129 (1.8%)
Risk factors for stroke	
Heart failure	1998 (27.7%)
Hypertension	4378 (60.6%)
Age (≥75 years)	2483 (34.4%)
Diabetes mellitus	1326 (18.4%)
Stroke/transient ischemic attack	991 (13.7%)
CHADS_2_ score	1.7 ± 1.2
CHA_2_DS_2_-VASc score	2.8 ± 1.6
HAS-BLED score (n = 6846)	1.5 ± 1.0
BP measurement times	14.6 ± 5.0
Systolic BP, mmHg	126.0 ± 16.1
SD, mmHg	11.0 ± 4.2
TTR (110–130 mmHg), %	49.5 ± 28.3
FIR (110–130 mmHg), %	50.6 ± 25.7
Diastolic BP, mmHg	73.3 ± 11.0
Heart rate, /min	72.4 ± 13.2
Creatinine clearance, mL/min (n = 5925)	68.5 ± 27.7
Hemoglobin, g/dL (n = 6398)	13.7 ± 1.7
Medications	
Warfarin	6269 (86.8%)
PT-INR (n = 6269)	1.91 ± 0.49
Time in therapeutic range*, % (n = 5934)	59.6 ± 28.8
Antiplatelet	1882 (26.0%)
Aspirin	1628 (22.5%)
Other antiplatelets	420 (5.8%)
Warfarin + antiplatelet	1329 (18.4%)
ARB/ACE-I	3850 (53.3%)
Na channel blockers	1452 (20.1%)
β-blockers	1128 (15.6%)
K channel blockers**	1011 (14.0%)
Ca channel blockers	476 (6.6%)
Digitalis	777 (10.8%)

Data are number of patients (%) or mean ± standard deviation (SD).

* Target PT-INR was 2.0–3.0 (<70 years) or 1.6–2.6 (≥70 years).

** Bepridil was classified as K channel blocker.

COPD, chronic obstructive pulmonary disease; CHADS_2_, congestive heart failure, hypertension, age ≥ 75 years, diabetes mellitus, and history of stroke or TIA; CHA_2_DS_2_-VASc, additionally, vascular disease (coronary artery disease), age 65–74 years, and female sex; HAS-BLED, hypertension (systolic BP ≥ 140 mmHg), abnormal renal/liver function, stroke, bleeding history or predisposition, labile INR (episodes of INR ≥ 3.5), elderly (age > 65 years), drugs (use of antiplatelets)/alcohol concomitantly; BP, blood pressure; TTR, time in target range; FIR, frequency in time; PT-INR, prothrombin time international normalized ratio; ARB, angiotensin II receptor blocker; ACE-I, angiotensin converting enzyme inhibitor.

### 3.2 Event rates and predictive ability of visit-to-visit BP indices for adverse events

During the 2-year follow-up period, thromboembolism, major hemorrhage, all-cause death, and cardiovascular death occurred in 110 (1.5%), 121 (1.7%), 168 (2.3%), and 60 (0.8%) patients, respectively. Corresponding incidence rates were 0.8, 0.8, 1.2, and 0.4 /100 person-years, respectively, during the follow-up period of 14,580 person-years.

**Fig. 3:**
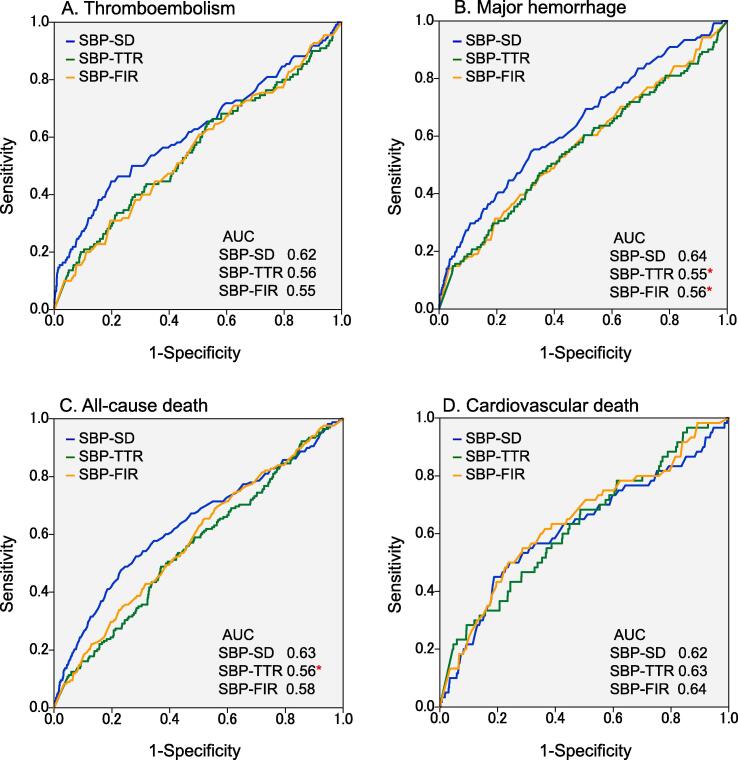
ROC curves of visit-to-visit SBP variability/consistency indices for thromboembolism (A), major hemorrhage (B), all-cause death (C), and cardiovascular death (D). ROC, receiver operating characteristic; SBP, systolic blood pressure; SD, standard deviation; TTR, time in target range; FIR, frequency in range; AUC, area under the ROC curve. Target SBP was 110–130 mmHg. * P < 0.05 vs. SBP-SD by the DeLong’s test.

ROC curves of visit-to visit SBP variability/consistency indices for adverse events are shown in Fig. 3. The AUCs of SBP-SD, SBP-TTR and SBP-FIR for thromboembolism, major hemorrhage, all-cause death, and cardiovascular death were 0.62, 0.64, 0.63, and 0.64 for SBP-SD; 0.56, 0.55, 0.56, and 0.56 for SBP-TTR; 0.55, 0.56, 0.58, and 0.57 for SBP-FIR; respectively. The AUCs of SBP-SD were over 0.6 for all adverse events and significantly larger than those of SBP-TTR for major hemorrhage (P = 0.010) and all-cause death (P = 0.014), and SBP-FIR for major hemorrhage (P = 0.016) (Table 2 and Fig. 3).

**Table 2:** Predictive ability of visit-to visit SBP indices for adverse events.

	**Thromboembolism**		**Major hemorrhage**		**All-cause death**		**Cardiovascular death**	
	AUC (95% CI)	*P*-value*	AUC (95% CI)	*P*-value*	AUC (95% CI)	*P*-value*	AUC (95% CI)	*P*-value*
**SBP-SD**	0.62 (0.56–0.68)	–	0.64 (0.59–0.69)	–	0.63 (0.59–0.68)	–	0.63 (0.54–0.70)	–
**SBP-TTR** **(110–130 mmHg)**	0.56 (0.50–0.61)	0.104	0.55 (0.50–0.61)	0.010	0.56 (0.51–0.60)	0.014	0.63 (0.55–0.70)	0.902
**SBP-FIR** **(110–130 mmHg)**	0.55 (0.50–0.61)	0.081	0.56 (0.51–0.62)	0.016	0.58 (0.54–0.62)	0.068	0.64 (0.57–0.72)	0.651

* Compared with SBP-SD by the DeLong’s test.

SBP, systolic blood pressure; AUC; area under the receiver-operating-characteristic curve; CI, confidence interval; TTR, time in target range; FIR, frequency in range; SD, standard deviation;

### 3.3 Factors associated with visit-to-visit BP variability/consistency

According to the multiple regression analyses, 13 variables including age, sex, creatinine clearance, baseline SBP, hemoglobin, warfarin dose, heart failure, hypertension, diabetes mellitus, stroke/TIA, and use of angiotensin II receptor blocker (ARB) or angiotensin converting enzyme inhibitor (ACE-I), β-blocker, and Ca channel blocker were identified as factors associated with SBP-SD (Supplementary Table 1). In contrast, 5 factors (body weight, baseline systolic and diastolic BP, warfarin dose, and ARB/ACE-I use) were significantly associated with SBP-TTR (Supplementary Table 2). Similarly, 5 factors (creatinine clearance, baseline systolic and diastolic BP, warfarin dose, and ARB/ACE-I use) were significantly associated with SBP-FIR (Supplementary Table 3).

## 4 Discussion

The major finding of this study was that the AUCs of SBP-SD were significantly larger than those of SBP-TTR for major hemorrhage and all-cause death in patients with NVAF.

### 4.1 Impact of BP variability and single BP measurement on adverse events

BP visit-to-visit variability [24], as an index of long-term BP variability [11], has been reportedly a risk factor for various clinical events and mortality in the general population and the patients with hypertension [25], [26], [27], [28], [29]. In patients with NVAF, the influence of BP visit-to-visit variability on adverse events was investigated in a substudy of the AFFIRM (Atrial Fibrillation Follow-Up Investigation of Rhythm Management) study [16] and our previous subanalysis of the J-RHYTHM Registry [12]. In both studies, larger SBP-SD was significantly associated with an increased risk of thromboembolism, major hemorrhage, and all-cause mortality [12], [16]. We further demonstrated that the predictive ability of SBP-SD for thromboembolism and all-cause death was comparable to that of SBP-end, whereas it was significantly superior to that of BP-end for major hemorrhage (AUCs of SBP-SD and SBP-end were 0.65 and 0.55, P = 0.012) and composite events (0.65 and 0.51, P < 0.001) [12].

These findings indicated that the BP visit-to-visit variability had stronger impact on the incidence of adverse events than a single BP measurement, especially for major hemorrhage. Thus, repetitive SBP fluctuation could be an ominous sign for subsequent adverse events in actual clinical setting.

### 4.2 Impact of BP variability and BP consistency on adverse events

Long-term BP consistency was also reportedly associated with all-cause mortality and major adverse cardiovascular events [30], [31]. In patients with NVAF, we reported that SBP-TTR of 110–130 mmHg, an index of BP consistency during the follow-up period, was associated with cardiovascular death in the previous *post hoc* analysis of the J-RHYTHM Registry [13]. However, SBP-TTR using the Rosendaal method [17] is calculated by a complex formula and generally requires a specific program or software. Therefore, we also calculated SBP-FIR, as an index of BP consistency, in the present study. This index was adopted in a subanalysis of the GARFIELD-AF (Global Anticoagulant Registry in the FIELD-Atrial Fibrillation) study [20], as an index of the quality of PT-INR control in patients receiving warfarin, and can be obtained by a simple formula in daily clinical practice. As shown in Table 2, there were no significant differences in the AUCs between SBP-TTR and SBP-FIR for all adverse events. Subsequently, the AUCs of SBP-TTR and SBP-FIR for adverse events were compared with those of SBP-SD in the present study. As shown in Table 2 and Fig. 3, the AUCs of SBP-SD were over 0.6 for all adverse events and significantly larger than those of SBP-TTR for major hemorrhage and all-cause death.

These findings indicated that the BP visit-to-visit variability had stronger impact on the incidence of adverse events than the BP consistency, especially for major hemorrhage and all-cause death. It seems reasonable because the indices of BP consistency (either SBP-TTR or SBP-FIR) do not include information of the range or degree (how high or how low) of BP fluctuation. For instance, among patients who measured BP on the same visit days during the follow-up period and had BP values out of the target range with the same frequency, patients with larger BP fluctuation could have higher risk of adverse events, despite the same SBP-TTR.

Accordingly, BP variability is more critical to predict the risk of future adverse events than BP consistency. Since adverse events often occur even in patients on warfarin with excellent time in therapeutic range of PT-INR, the concept of BP variability/consistency from the present study might be extrapolated to PT-INR variability/consistency. Indeed, our previous report revealed that variability of anticoagulation intensity with warfarin (PT-INR-SD) was significantly correlated with BP variability (SBP-SD) [12].

### 4.3 Factors associated with visit-to-visit BP variability/consistency

Multiple regression analyses revealed that number of factors associated with BP variability (SBP-SD) was larger than that with BP consistency (SBP-TTR and SBP-FIR). Various clinical factors were independently associated with BP variability rather than BP consistency. Therefore, it seems reasonable that BP variability could be used as a more powerful indicator of adverse events than BP consistency. Interestingly, Ca channel blocker use was significantly associated with less BP variability.

### 4.4 Limitations

This study had several limitations. First, it was a *post hoc* analysis of data from the J-RHYTHM Registry [15], [23] and was therefore hypothesis-generating in nature. Second, study subjects were recruited from only 158 institutions in Japan in 2009 and most of the participating physicians specialized in cardiology and in the management of cardiac arrhythmias. Oral anticoagulant used was only warfarin. Therefore, these results may not be generalizable to the contemporary overall Japanese population with NVAF in the era of NOACs. In addition, since all study subjects were Japanese in this study, these data may not necessarily be applicable to other racial/ethnic populations. Third, BP measurement methods were not standardized. BP values were obtained by the auscultatory method or an automated sphygmomanometer, as appropriate for daily clinical practice in each institution. Patients with BP measurements < 4 times were excluded from the present subanalysis as in previous studies [12], [13], [16]. After exclusion of these patients (n = 180), however, event rates did not differ between the remaining patients (n = 7226) who were study subjects for the present subanalysis and the entire cohort of patients (n = 7406) for the main analysis [23]. Fourth, changes in antihypertensive drugs and dosage, and adherence to drugs during the follow-up period were not considered in the analysis. Finally, since the study design was not a randomized controlled trial, causality between visit-to-visit BP variability/consistency indices and adverse events could not be determined.

## 5 Conclusions

Among visit-to-visit BP variability and consistency indices, the predictive ability of SBP-SD for adverse events was superior to that of SBP-TTR and SBP-FIR, especially for major hemorrhage and all-cause death, in patients with NVAF. BP variability is a more powerful indicator of adverse events than BP consistency.

## 6 Authors’ contribution

Dr. Kodani contributed to the statistical analyses and wrote the manuscript. Drs. Inoue, Atarashi, Okumura, and Yamashita are on the Executive Committee of the J-RHYTHM Registry. They planned and supervised this study comprehensively and edited the manuscript, as appropriate. Dr. Suzuki calculated the BP-TTR. Dr. Origasa contributed as a statistical advisor.

## Disclosures

Dr. Kodani received remuneration from Daiichi-Sankyo; Dr. Inoue received remuneration from Boehringer Ingelheim, Bristol-Myers Squibb, and Daiichi-Sankyo; Dr. Atarashi received remuneration from Daiichi-Sankyo; Dr. Okumura received remuneration from Boehringer Ingelheim, Bristol-Myers Squibb, Daiichi-Sankyo, Johnson & Johnson, and Medtronic; Dr. Suzuki received research funding from Daiichi-Sankyo and Mitsubishi-Tanabe and remuneration from Bristol-Myers Squibb and Daiichi-Sankyo; and Dr. Yamashita received research funding from Bayer Healthcare, Bristol-Meyers Squibb, and Daiichi-Sankyo and remuneration from Bayer Healthcare, Bristol-Myers Squibb, Daiichi-Sankyo, Novartis, Ono Pharmaceutical, Otsuka Pharmaceutical, and Toa Eiyo.

## Declaration of Competing Interest

The authors declare that they have no known competing financial interests or personal relationships that could have appeared to influence the work reported in this paper.
