# Supplementary material for: Predictive ability of visit-to-visit blood pressure indices for adverse events in patients with non-valvular atrial fibrillation: Subanalysis of the J-RHYTHM Registry
Source: Int J Cardiol Heart Vasc. 2023 May 6;46:101216. doi: 10.1016/j.ijcha.2023.101216 (PMC10189411; doi:10.1016/j.ijcha.2023.101216)
Supplement: Supplementary data 1 [file mmc1.pdf]

Supplementary Materials

Predictive ability of visit-to-visit blood pressure indices for adverse events in patients with non-valvular atrial fibrillation: Subanalysis of the J-RHYTHM Registry

Eitaro Kodani, Hiroshi Inoue, Hirotsugu Atarashi, Ken Okumura, Shinya Suzuki, Takeshi Yamashita, Hideki Origasa, on behalf of the J-RHYTHM Registry Investigators

1. Supplementary Table 1. Factors associated with systolic BP-SD
2. Supplementary Table 2. Factors associated with systolic BP-TTR (110–130 mmHg)
3. Supplementary Table 3. Factors associated with systolic BP-FIR (110–130 mmHg)

Supplementary Table 1. Factors associated with systolic BP-SD

	Simple		Multiple**	
**Variable***	β (95% CI)	P value	β (95% CI)	P value
Age (per 1 year)	0.06 (0.05, 0.07)	<0.001	0.02 (0.01, 0.04)	0.006
Sex (women)	0.55 (0.34, 0.76)	<0.001	0.28 (0.01, 0.54)	0.040
Body weight (per 1 kg)	–0.28 (–0.04, –0.02)	<0.001	-	-
CrCl (per 1 mL/min)	–0.02 (–0.03, –0.02)	<0.001	–0.01 (-0.01, –0.00)	0.003
Systolic BP (per 1 mmHg)	0.05 (0.04, 0.05)	<0.001	0.04 (0.03, 0.05)	<0.001
Diastolic BP (per 1 mmHg)	0.01 (0.00, 0.01)	0.034	-	-
Hemoglobin (per 1 g/dL)	–0.27 (–0.33, –0.21)	<0.001	–0.10 (–0.18, –0.03)	0.005
Warfarin dose (per 1 mg)	–0.27 (–0.36, –0.19)	<0.001	–0.11 (–0.21, –0.01)	0.034
Heart failure	0.48 (0.26, 0.69)	<0.001	0.34 (0.08, 0.59)	0.010
Hypertension	1.05 (0.85, 1.24)	<0.001	0.28 (0.00, 0.55)	0.047
Diabetes mellitus	0.59 (0.34, 0.83)	<0.001	0.39 (0.11, 0.67)	0.007
Stroke/TIA	0.83 (0.56, 1.11)	<0.001	0.66 (0.35, 0.97)	<0.001
Antiplatelet use	0.41 (0.19, 0.63)	<0.001	-	-
ARB/ACE-I use	0.87 (0.68, 1.06)	<0.001	0.42 (0.16, 0.68)	0.001
Na channel blocker use	–0.25 (–0.50, –0.01)	0.041	-	-
β-blocker use	0.28 (0.01, 0.55)	0.041	0.41 (0.11, 0.70)	0.006
Ca channel blocker use	–0.46 (–0.85, –0.07)	0.022	–0.54 (–0.97, –0.11)	0.014

* Non-significant variables in simple regression analysis are not shown.
** Stepwise forward procedure using variables with P <0.05 in simple regression analysis.
BP, blood pressure; SD, standard deviation; β, regression coefficient; CI, confidence interval; CrCl, creatinine clearance; TIA, transient ischemic attack, ARB, angiotensin II receptor blocker; ACE-I, angiotensin converting enzyme inhibitor.

Supplementary Table 2. Factors associated with systolic BP-TTR (110–130 mmHg)

	Simple		Multiple**	
**Variable***	β (95 % CI)	P value	β (95% CI)	P value
Age (per 1 year)	–0.20 (–0.27, –0.14)	<0.001	-	-
Sex (women)	–2.78 (–4.22, –1.35)	<0.001	-	-
Body weight (per 1 kg)	0.10 (0.04, 0.15)	<0.001	0.08 (0.02, 0.14)	0.011
CrCl (per 1 mL/min)	0.06 (0.04, 0.09)	<0.001	-	-
Systolic BP (per 1 mmHg)	–0.45 (–0.49, –0.41)	<0.001	–0.39 (–0.44, –0.34)	<0.001
Diastolic BP (per 1 mmHg)	–0.09 (–0.12, –0.05)	<0.001	0.07 (0.02, 0.12)	0.008
Heart rate (per 1 bpm)	–0.05 (–0.10, –0.00)	0.035	-	-
Hemoglobin (per 1 g/dL)	0.48 (0.08, 0.87)	0.019	-	-
Warfarin dose (per 1 mg)	1.01 (0.42, 1.61)	0.001	1.04 (0.38, 1.71)	0.002
Hypertension	–6.80 (–8.12, –5.47)	<0.001	-	-
Diabetes mellitus	–1.97 (–3.66, –0.29)	0.022	-	-
ARB/ACE-I use	–6.37 (–7.67, –5.07)	<0.001	–5.15 (–6.66, –3.64)	<0.001

* Non-significant variables in simple regression analysis are not shown.

** Stepwise forward procedure using variables with P <0.05 in simple regression analysis.

BP, blood pressure; TTR, time in target range; β, regression coefficient; CI, confidence interval; CrCl, creatinine clearance; ARB, angiotensin II receptor blocker; ACE-I, angiotensin converting enzyme inhibitor.

Supplementary Table 3. Factors associated with systolic BP-FIR (110–130 mmHg)

	Simple		Multiple**	
**Variable***	β (95 % CI)	P value	β (95% CI)	P value
Age (per 1 year)	–0.20 (–0.26, –0.14)	<0.001	-	-
Sex (women)	–2.58 (–3.88, –1.28)	<0.001	-	-
Body weight (per 1 kg)	0.09 (0.05, 0.14)	<0.001	-	-
CrCl (per 1 mL/min)	0.06 (0.04, 0.09)	<0.001	0.04 (0.02, 0.07)	0.002
Systolic BP (per 1 mmHg)	–0.42 (–0.45, –0.38)	<0.001	–0.37 (–0.42, –0.33)	<0.001
Diastolic BP (per 1 mmHg)	–0.08 (–0.11, –0.04)	<0.001	0.07 (0.02, 0.11)	0.004
Heart rate (per 1 bpm)	–0.05 (–0.09, –0.00)	0.044	-	-
Hemoglobin (per 1 g/dL)	0.51 (0.15, 0.87)	0.006	-	-
Warfarin dose (per 1 mg)	0.84 (0.30, 1.38)	0.002	0.78 (0.17, 1.40)	0.012
Hypertension	–6.35 (–8.12, –5.14)	<0.001	-	-
Diabetes mellitus	–1.89 (–3.43, –0.36)	0.015	-	-
ARB/ACE-I use	–6.37 (–7.67, –5.07)	<0.001	–4.46 (–5.81, –3.10)	<0.001

* Non-significant variables in simple regression analysis are not shown.
** Stepwise forward procedure using variables with P <0.05 in simple regression analysis.
BP, blood pressure; FIR, frequency in range; β, regression coefficient; CI, confidence interval; CrCl, creatinine clearance; ARB, angiotensin II receptor blocker; ACE-I, angiotensin converting enzyme inhibitor.
